# Pathogenesis of the *Candida* *parapsilosis* Complex in the Model Host *Caenorhabditis elegans*

**DOI:** 10.3390/genes9080401

**Published:** 2018-08-08

**Authors:** Ana Carolina Remondi Souza, Beth Burgwyn Fuchs, Viviane de Souza Alves, Elamparithi Jayamani, Arnaldo Lopes Colombo, Eleftherios Mylonakis

**Affiliations:** 1 Special Mycology Laboratory, Division of Infectious Diseases, Federal University of São Paulo-UNIFESP, 04039-032 São Paulo, SP, Brazil; carolina.remondi@yahoo.com.br (A.C.R.S.); arnaldolcolombo@gmail.com (A.L.C.); 2 Division of Infectious Diseases, Rhode Island Hospital, Alpert Medical School of Brown University, Providence, RI 02903, USA; ejayamani@partners.org; 3 Microorganisms Cell Biology Laboratory, Microbiology Department, Biological Sciences Institute, Federal University of Minas Gerais, Belo Horizonte 31270-901, MG, Brazil; gouveiava@ufmg.br

**Keywords:** *Candidaparapsilosis*, *Caenorhabditiselegans*, hyphae, invertebrate infection model, host-pathogen interaction

## Abstract

*Caenorhabditiselegans* is a valuable tool as an infection model toward the study of *Candida* species. In this work, we endeavored to develop a *C*. *elegans*-*Candida* *parapsilosis* infection model by using the fungi as a food source. Three species of the *C. parapsilosis* complex (*C. parapsilosis* (*sensu stricto*), *Candida orthopsilosis* and *Candida metapsilosis*) caused infection resulting in *C. elegans* killing. All three strains that comprised the complex significantly diminished the nematode lifespan, indicating the virulence of the pathogens against the host. The infection process included invasion of the intestine and vulva which resulted in organ protrusion and hyphae formation. Importantly, hyphae formation at the vulva opening was not previously reported in *C*. *elegans*-*Candida* infections. Fungal infected worms in the liquid assay were susceptible to fluconazole and caspofungin and could be found to mount an immune response mediated through increased expression of *cnc*-*4*, *cnc*-*7*, and *fipr-22*/*23*. Overall, the *C*. *elegans*-*C*. *parapsilosis* infection model can be used to model *C*. *parapsilosis* host-pathogen interactions.

## 1. Introduction

*Candida parapsilosis* is a common human opportunistic pathogen able to cause superficial and invasive diseases. Most notably, it causes bloodstream infections (BSIs) in very low birth weight neonates and in patients with catheter-associated candidemia and/or intravenous hyperalimentation [1,2,3]. In 2005, the genetically heterogeneous taxon *C*. *parapsilosis* was reclassified into three species: *C*. *parapsilosis* (*sensu stricto*), *Candida orthopsilosis*, and *Candida metapsilosis* [4]. As of yet, it is unclear if there are putative differences between virulence traits among species within the *C*. *parapsilosis* complex [5,6,7]. *C*. *parapsilosis* (*sensu lato*) is the most common non-*albicans Candida* species (NAC) isolated from BSIs in Spain, Italy, many countries in Latin America, while being described as prevalent in U.S. medical centers [8,9,10,11].

Over the past decade, invertebrate models have become increasingly valuable to facilitate the study of fungal pathogenesis [12]. Several factors triggered the development of these models, including ethical issues, costs, and physiological simplicity. Moreover, the innate immune mechanisms between invertebrates and mammals share evolutionary conservation, which provides insight into common virulence factors involved in fungal pathogenesis of different types of hosts [13,14,15,16,17].

In particular, *Caenorhabditis elegans* has been used successfully as a candidiasis infection model [18], and its utility has been demonstrated in the assessment of fungal virulence traits and identification of new anti-fungal compounds [18,19,20]. Nematodes consume fungal pathogens, substituting for the normal laboratory diet, *Escherichia coli*. The ingested fungi establish an infection within the worm gut that can be characterized by the accumulation of yeast and distention of the intestine. The infected nematodes can be followed with either solid or liquid media assay conditions. In liquid medium assays, yeast form hyphae that protrude through the worm cuticle [18,21]. Both *Candida albicans* and non-*albicans* species have been found to cause lethal infections in *C*. *elegans* [18].

Although *C*. *elegans* has proven to be a valuable host to study *C*. *albicans* and a limited number of non-*albicans* species, there are still limited evaluations applied to the study of *C*. *parapsilosis* species complex infections [22]. In this study, we developed a *C*. *parapsilosis* (*sensu lato*)-*C*. *elegans* infection model and demonstrated the utility of this model to study virulence traits of this pathogenic yeast. Furthermore, our endeavors provide insight into the host’s defense mechanisms involved against *C*. *parapsilosis* infection. We describe the reduced lifespan of worms that ingest *C*. *parapsilosis* and the host symptoms that follow, which differ from those involved in *C*. *albicans*-*C*. *elegans* infection.

## 2. Materials and Methods

### 2.1. Strains and Media

The *Candida* strains used in these experiments were obtained from the American Type Culture Collection (ATCC) and included: *C*. *parapsilosis* (*sensu stricto*) ATCC 22019, *C*. *orthopsilosis* ATCC 96141, and *C*. *metapsilosis* ATCC 96143. The *C*. *elegans* strains described in this study were: *N2* bristol [23], *glp*-*4*; *sek*-*1* [24] and *pmk*-*1* [24] (Table 1). The *C*. *elegans* strains were maintained at 25 °C (*N2* and *pmk-1*) and 15 °C (*glp*-*4*; *sek*-*1*) and propagated on Nematode Growth Medium (NGM) agar plates seeded with the *E*. *coli* strain HB101 using established procedures [23]. Yeast cultures were grown in yeast extract, peptone, dextrose (YPD) medium at 30 °C. *E*. *coli* (HB101) was grown in Luria Broth (LB, Sigma Aldrich, Saint Louis, MO, USA) at 37 °C.

### 2.2. Caenorhabditis elegans Liquid Medium Killing Assays

The infection assay was performed as previously described [14]. In brief, worms grown on NGM plates were washed with M9 buffer and placed on 24 h old *C*. *parapsilosis* species complex lawns (on brain heart infusion (BHI) agar plates) for 4 h. After this, the worms were washed off the plates and transferred to wells (*n* = 30 per well) in a twelve-well plate that contained 2 mL of liquid medium (20% BHI, 80% M9, 45 μg/mL Kan). The plates were incubated at 25 °C and nematode survival was examined at 24 h intervals for the subsequent 144 h.

### 2.3. Antifungal Drug Treatments

To study the efficacy of antifungal agents against the *C*. *parapsilosis* complex in this model, fluconazole (Sigma Aldrich) and caspofungin (Merck, Kenilworth, NJ, USA) were dissolved in dimethyl sulphoxide (DMSO) and added to the liquid assay. The exposure was relative to the minimum inhibitory concentration (MIC) (Table 2): 1×MIC, 2×MIC, and 0.5×MIC. Therefore, for fluconazole, the following concentrations were tested: 1.0 µg/mL (1×), 2.0 µg/mL (2×) and 0.5 µg/mL (0.5×). For caspofungin, the concentrations tested were: 0.5 µg/mL (1×), 1 µg/mL (2×) and 0.25 µg/mL (0.5×) (Table 2). Worms were incubated at 25 °C and survival was monitored daily [18]. Worms were considered dead when they failed to respond to the touch of a platinum wire pick [14].

### 2.4. Microscopic Studies

To study *C*. *parapsilosis* colonization in *C*. *elegans*, *glp*-*4*, *sek*-*1* nematodes were pre-infected with *C*. *parapsilosis* reference strains for 4 h at 25 °C [14]. Then, the worms were washed three times in M9 buffer and transferred to fresh BHI:M9 medium and incubated at 25 °C for 20 and 48 h. The worms were paralyzed with 1 mM sodium azide solution and placed on 2% agarose pads to capture images at 20 and 48 h post infection [25]. A confocal laser microscope was used for observation (Carl Zeiss M1, Oberkochen, Germany).

### 2.5. Quantitative RT-PCR Analyses of Candida parapsilosis Infected Nematodes

Following infection, N2 worms were treated and RNA was extracted as previously described [26]. The sample quality was assessed through RNA concentration and the 260/280 or 260/230 ratios using a Nanovue spectrophotometer (GE LifeSciences, Piscataway, NJ, USA).

RNA was reverse transcribed to cDNA using the Verso cDNA Synthesis Kit (Thermo Scientific, Waltham, MA, USA). cDNA was analyzed by quantitative real-time (qRT-PCR) using iTaq Universal SYBR Green Supermix^®^ (Bio-Rad, Hercules, CA, USA) at CFX1000 machine (Bio-Rad) and specific primers to the following targets: *Fipr22*/*23*, *abf*-*1*, *abf*-*2*, *cnc*-*4*, and *cnc*-*7* (Table 3). All values were normalized against the reference gene *act-1* [19,27,28,29,30]. The thermal cycling conditions were comprised of an initial step at 95 °C for 30 s, followed by thirty-five cycles involving denaturation at 95 °C for 5 s, annealing at 58 °C for 15 s and extension at 72 °C for 1 min. The 2^−ΔΔCt^ was calculated for relative quantification of gene expression.

### 2.6. Statistics

Killing curves were plotted and the estimation of differences in survival (log-rank and Wilcoxon tests) was performed by the Kaplan-Meier method using GraphPad Prism 5 (GraphPad Software, La Jolla, CA, USA). A *p*-value of <0.05 was considered significant. Relative gene expression was compared using Bonferroni’s Method with GraphPad Prism 5 (GraphPad Software). Each experiment was repeated at least three times, and each independent experiment gave similar results.

## 3. Results

### 3.1. Killing Caenorhabditis elegans by Candida parapsilosis Species Complex

First, we assessed the ability of different species within the *C. parapsilosis* complex to cause infection. The results showed that all three species (*C. parapsilosis* (*sensu stricto*), *C. orthopsilosis* and *C. metapsilosis*) were able to kill *C. elegans*. More specifically, in triplicate experiments, we found that *C. parapsilosis* (*sensu stricto*) 22019, *C. orthopsilosis* 96141 and *C. metapsilosis* 96143 killed *C. elegans* with the time to 50% mortality ranging from four to six days for *C. parapsilosis* (*sensu stricto*), *C. orthopsilosis* and *C. metapsilosis*. In all cases, mortality was higher than the *E. coli* (HB101) control group (*p*-value = 0.003 for *C. parapsilosis* (*sensu stricto*); *p*-value = 0.009 for *C. orthopsilosis* and *p*-value = 0.008 for *C. metapsilosis*). Interestingly, there was no significant difference between the *C. parapsilosis* species complex infection groups.

### 3.2. Hyphal Formation of Candida parapsilosis Species Complex within Caenorhabditis elegans

**Figure 1:**
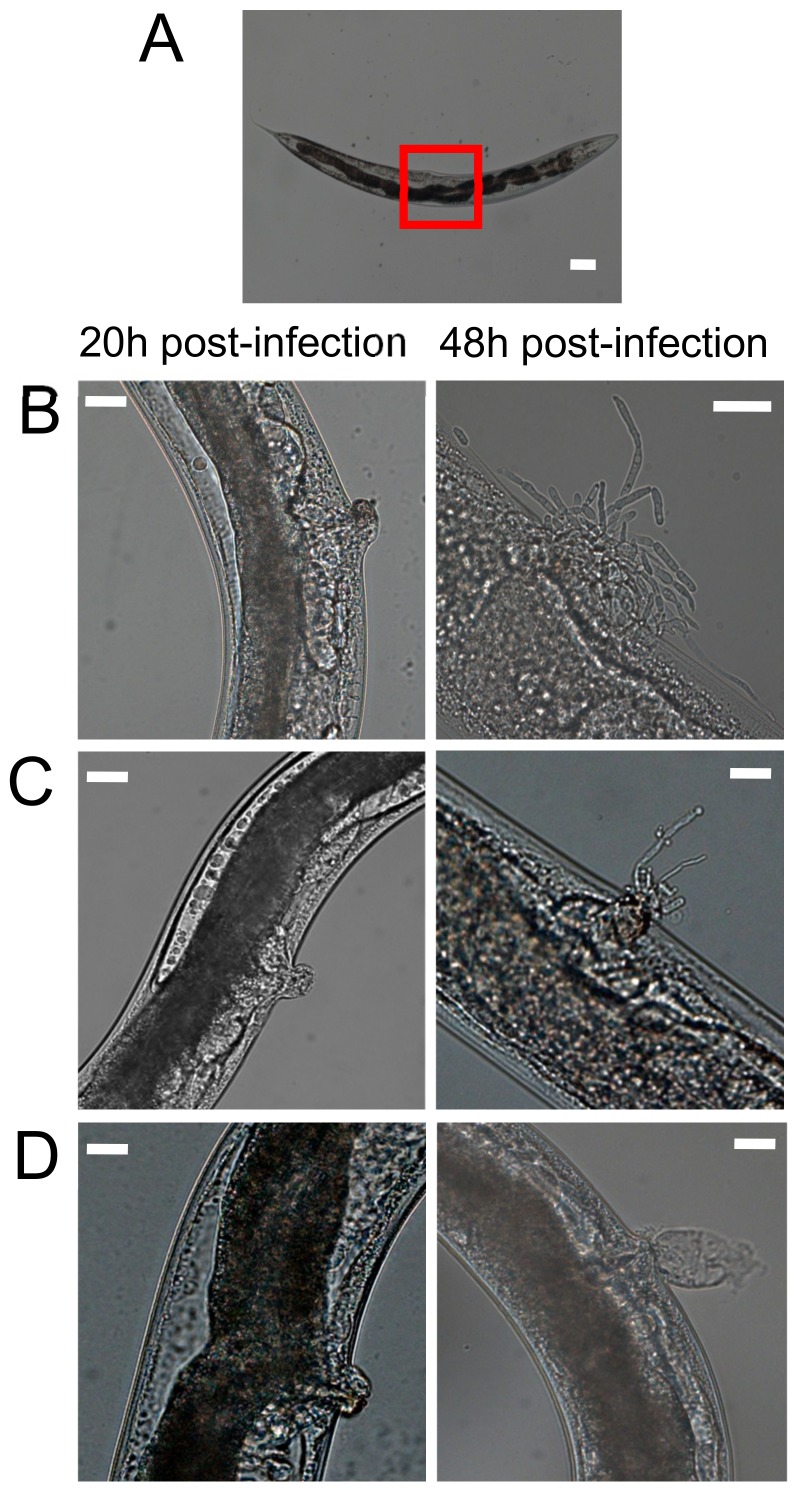
*C. elegans* physiological effects after *C*. *parapsilosis* exposure. (**A**) *Escherichia coli* exposure shows no adverse effects. The vulva region is highlighted in red; (**B**) *C. parapsilosis* (*sensu stricto*); (**C**) *C. orthopsilosis*; (**D**) *C. metapsilosis.* Scale Bar-20 µm.

*Candida albicans* directed killing of *C*. *elegans* host is characterized by the formation of filaments that pierce the worm cuticle in a liquid media assay [18]. We investigated whether filaments could be observed in the *C*. *parapsilosis*-*C*. *elegans* infection model. Nematode morphology of the infected worms was observed at 20 h and 48 h post-infection with the reference strains ATCC22019, ATCC96141, and ATCC96143. Worms that consumed *E. coli* appeared in good health (Figure 1A). As shown in Figure 1, the progress of infection and death of *C. elegans* was similar when they were infected by *C. parapsilosis* (*sensu stricto*) (Figure 1B), *C. orthopsilosis* (Figure 1C) or *C. metapsilosis* (Figure 1D). The intestine was distended after ingesting fungal cells (Figure 1B–D), and hyphae start to accumulate within the intestines of live animals 20 h after infection. Filaments were observed breaching the worm at the vulva by 48 h and were observed fully protruding only in lethally infected nematodes.

### 3.3. Treatment with Antifungal Drugs

**Figure 2:**
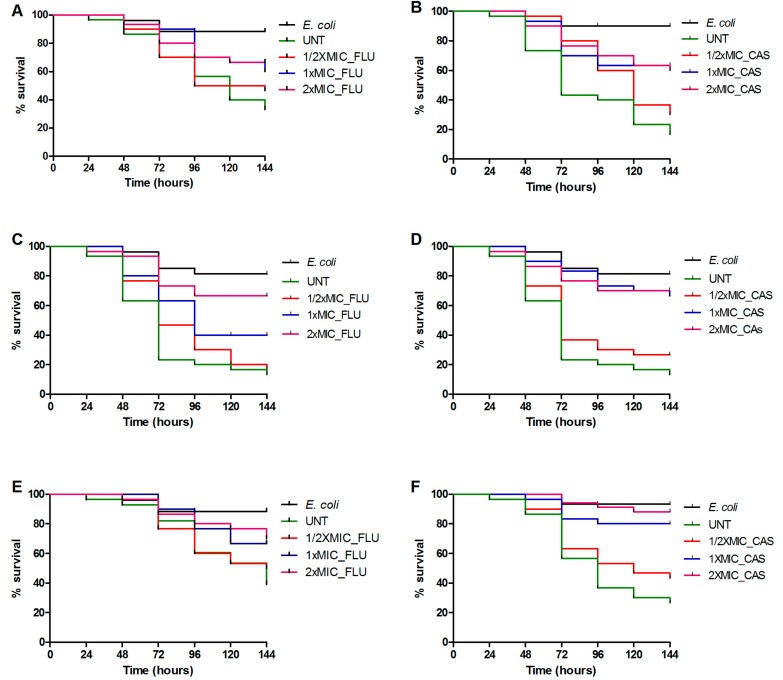
Efficacy of fluconazole (FLU) and caspofungin (CAS) during *C. elegans* infection with *C. parapsilosis* (*sensu stricto*), *C. orthopsilosis* and *C. metapsilosis* reference strains. (**A**) *C. parapsilosis* ATCC 22019, FLU treatment. *p* < 0.05 to FLU_1×MIC. (**B**) *C. parapsilosis* ATCC 22019, CAS treatment. *p* = 0.0004 to CAS_1×MIC. (**C**) *C. orthopsilosis* ATCC 96141, FLU treatment. *p* < 0.007 to FLU_1×MIC. (**D**) *C. orthopsilosis* ATCC 96141, CAS treatment. *p* = 0.0001 to CAS_1×MIC. (**E**) *C. metapsilosis* ATCC 96143, FLU treatment. *p* < 0.05 to FLU_1×MIC. (**D**) *C. metapsilosis* ATCC 96143, CAS treatment. *p* < 0.0001 to CAS_1×MIC.

To investigate if compounds could inhibit the fungal infection, *C. elegans* were challenged with *C. parapsilosis* (*sensu stricto*), *C. orthopsilosis*, or *C. metapsilosis* by ingesting the three investigational strains individually on solid media and were then transferred to liquid media, where they were treated with fluconazole and caspofungin. As demonstrated in Figure 2, we observed a dose dependent prolonged survival in response to caspofungin or fluconazole, so that the wells containing antifungal at a concentration below the effective dose (0.5×MIC) resulted in dead worms, similar to those observed in wells that contained no antifungal. On the other hand, a statistically significant increase in survival (*p* < 0.001) was detected when the worms were treated with caspofungin and fluconazole in concentrations at 1×MIC and 2×MIC. In fact, on average, after administration for 144 days, 1×MIC and 2×MIC of fluconazole allowed for, at least, 57% of the nematodes to survive, while 0.5×MIC of this azole resulted in only 38% of the worms being alive. Similarly, caspofungin treatment at 0.5×MIC resulted in 31% nematode survival, whereas at a dose of 1×MIC and 2×MIC, this increased to 69% and 74%, respectively.

### 3.4. Caenorhabditis elegans Immune Response to Candida parapsilosis Complex Infection

**Figure 3:**
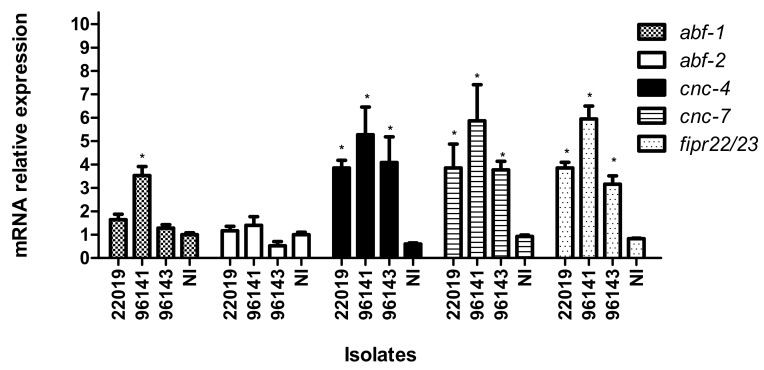
Relative expression of antimicrobial peptides after infection with *C*. *parapsilosis* (*sensu stricto*) (ATCC 22019), *C*. *orthopsilosis* (ATCC 96141), *C*. *metapsilosis* (ATCC 96143), and in non-infected worms (NI). Data are presented as the average of three biological replicates each normalized to a control gene. The error bars represent the standard errors of the mean for three independent biological replicates. * *p* < 0.005.

**Figure 4:**
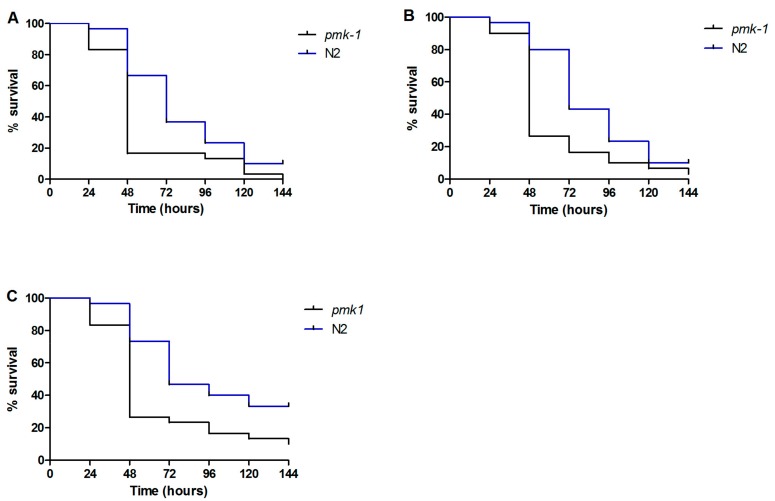
Infection assay with *C. elegans* wild-type (N2) and *pmk-1* animals shows that *pmk-1* was more susceptible to (**A**) *C. parapsilosis* (*sensu stricto*) (ATCC 22019), (**B**) *C. orthopsilosis* (ATCC 96141) and (**C**) *C. metapsilosis* (ATCC 96143) infection (*p* < 0.05).

In order to understand the defense mechanisms involved against *C. parapsilosis* infection, we focused our attention on five antimicrobial peptides (AMP), which are postulated to have antifungal activity in vivo [19,31]. As shown in Figure 3, 4 h after exposure to *C. parapsilosis* (*sensu stricto*), *C. orthopsilosis*, and *C. metapsilosis,* the expression of the AMP’s *cnc-4*, *cnc-7*, and *fipr22/23* increased significantly in response to the presence of the fungal pathogens compared to an *E. coli* control group. The expression of *abf-1* increased only when *C. elegans* was challenged with *C. orthopsilosis* and the *abf-2* expression was unchanged for any of the species involved. Corroborating these findings, we found that *C. elegans pmk-1* (*km25*) mutants were hyper-susceptible to infection with *C. parapsilosis* complex (*p* < 0.05). The PMK-1 mitogen-activated protein (MAP) kinase, orthologous to the mammalian p38 MAPK, in *C. elegans* immunity, is a central regulator of nematode defenses and is required for the basal and pathogen-induced expression of three antifungal immune effectors (*cnc-4*, *cnc-7* and *fipr22/23*), but not *abf-2* (Figure 4).

## 4. Discussion

In this study, the *C*. *elegans* invertebrate infection model emerges as a valuable tool for the study of *C. parapsilosis*. Using reference strains, we revealed that *C. parapsilosis* species complex cells, ingested by *C. elegans,* infect and kill the nematode. These data corroborate previous studies showing that *C. orthopsilosis* and *C. metapsilosis* are capable of causing invasive infection in mouse models and in humans [8,32,33].

Although the pathogenic potential of the three *C. parapsilosis* complex species is characterized, little is known about the putative differences of their virulence. Previous studies have reported that *C. metapsilosis* seems to be the least virulent member of the *C. parapsilosis* complex in both in vitro and in vivo assays [17,34,35,36]. However, there is still some controversy within the topic. In a study by Treviño-Rangel et al. (2014), the authors suggest that the three species of the *C. parapsilosis* group possess a similar pathogenic potential in disseminated candidiasis [32]. Corroborating these data, we found that, in *C. elegans* infection model, *C. metapsilosis* had similar mortality rates to that of *C. parapsilosis* (*sensu stricto*) and *C. orthopsilosis*.

*Candida* hyphal formation is a key virulence determinant that allows cells to invade host tissues and escape phagocytic destruction [37,38]. Pukkila-Worley et al. (2009) demonstrated that the switching from budding yeast to a filamentous (hyphal) form results in aggressive tissue destruction and death of the nematode [21]. In this context, we investigated if this change also took place during infection by *C. parapsilosis*. As expected, all three *C. parapsilosis* strains produced filaments and this phenomenon seems to be associated with *C. elegans* killing. The *C. elegans*-*C. albicans* infection model revealed that *C. albicans* infections within this host begin to accumulate hyphae at the upper part of the gut at the initial stages of the infection process that then spread to consume the entire worm [21]. By contrast, in *C*. *elegans* infected with *C*. *parapsilosis* species complex filaments initiated at the vulva rather than the gut, indicating differences in the *C*. *elegans*-*C*. *albicans* versus *C*. *elegans*-*C*. *parapsilosis* infection processes and host-pathogen interactions.

Although the pathogenicity process may be altered between the two host-pathogen infection models, a response to drug treatment remains conserved. Different studies have demonstrated the utility of invertebrate infection models, including *C. elegans*, as a screening method for potential antifungal compounds [18,25]. In this study, we found a correlation between the in vivo efficacy of antifungals during *C. parapsilosis* (*sensu stricto*), *C. orthopsilosis*, and *C. metapsilosis* infection and their in vitro susceptibility profiles for the standard care therapeutic agents, fluconazole and caspofungin, in a dose dependent manner. By demonstrating that both therapeutic agents have a protective effect during infection in the *C. elegans* model, we gave evidence that drug discovery assays applied to other *Candida* spp.-*C*. *elegans* models are potentially applicable.

As a whole organism, the model also yields the ability to investigate host immune responses to the pathogen. The nematode *C. elegans* is able to specifically recognize and defend itself against bacterial and fungal pathogens due the presence of complex, inducible, antimicrobial, innate immune responses, which involve the activation of antifungal effectors and core immune genes [19,31,39,40,41,42]. Pukkila-Worley et al. (2011) showed that exposure to *C. albicans* stimulated a rapid host response involving approximately 1.6% of the genome, with the majority of the genes encoding antimicrobial, secreted, or detoxification proteins [19]. We therefore used qPCR to check the expression of five AMP genes (*abf-1*, *abf-2*, *cnc-4*, *cnc-7*, and *fipr22/23*) and two transcriptional factors (*zip-2* and *atf-7*). The *abf-1* and *abf-*2 genes belong to a family of six genes encoding antibacterial factors (ABFs) in *C. elegans* and their antimicrobial action were previously described [19,31,43,44]. Expression of these two ABFs seems to be species-specific [41,45]. We found induction of *abf-1* only after *C. orthopsilosis* infection. Regarding *abf-2*, no expression was observed for any of the species.

The second family of AMP we evaluated was the caenacins (CNCs), which are expressed in the *C. elegans* epidermis and, therefore, play a direct role against pathogens that infect worms via the intestinal lumen or cuticle [41,46,47,48]. Induction of *cnc-4* and *cnc-7* after *C. albicans* has been previously described [41]. Accordingly, we found that the expression of both genes increases significantly in worms fed with any of the three *C. parapsilosis* species, when compared to an *E. coli* control.

In 2008, Pujol et al. described a group of uncharacterized genes that seem to be specifically induced upon fungal infection and could potentially encode AMPs [47]. These genes were called fungus-induced peptide related (*fipr*). During infection with *C. albicans*, the expression of *fipr22/23* is up-regulated [19]. In our study, we also observed induction of the *fipr22/23* gene by the three species belonging to the *C. parapsilosis* complex, suggesting that the FIPRs are involved in the defense mechanisms against *C. parapsilosis* infection. Taken together, our data demonstrated that *C. elegans* mounts a specific defense response against the three different species of *C. parapsilosis* complex.

## 5. Conclusions

In summary, we demonstrated that *C. elegans* can be used as an appropriate infection model to study the pathogenicity of *C. parapsilosis* (*sensu stricto*), *C. orthopsilosis* and *C. metapsilosis*, not only for evaluating the virulence traits of these species, but also to screen antifungal agents, and study mechanisms of innate immune response against these yeasts. Future studies should expand the model described here to yield more insights about the pathogenicity of these species, especially *C. orthopsilosis* and *C. metapsilosis*.

## Figures and Tables

**Table 1:** *Candida parapslosis* complex and *Caenorhabditis elegans* strains used in this study.

Strain	Description	Purpose	Reference
***Candida* species**			
*C. parapsilosis* (*sensu stricto*) ATCC 22019	WT ^a^	All experiments	ATCC
*Candida orthopsilosis* ATCC 96141	WT	All experiments	ATCC
*Candida metapsilosis* ATCC 93143	WT	All experiments	ATCC
***C. elegans***			
*N2*	WT	Immunity response	[23]
*glp-4*; *sek-1*	*glp-4(bn2) I*; *sek-1(km4)*	Killing assay, treatment with antifungal drugs, microscopic studies	[24]
*pmk-1*	*pmk-1(km25)*	Immunity response	[24]

^a^ Wild-type.

**Table 2:** In vitro activity against *C. parapsilosis* species complex reference strains.

	MIC (µg/mL)	
Strain	Fluconazole	Caspofungin
ATCC 22019	1.0	0.5
ATCC 96141	1.0	0.5
ATCC 96143	1.0	0.5

MIC: minimum inhibitory concentration.

**Table 3:** Oligonucleotide sequences used in this study.

Oligonucleotide ^a^	Sequence 5′ to 3′	Reference
ABF-1/Fw	CTGCCTTCTCCTTGTTCTCCTACT	[19]
ABF-1/Rv	CCTCTGCATTACCGGAACATC	[19]
ABF-2/Fw	TTTCCTTGCACTTCTCCTGG	This study ^b^
ABF-2/Rv	CGGTTCCACAGTTTTGCATAC	This study
CNC-4/Fw	ACAATGGGGCTACGGTCCATAT	This study
CNC-4/Rv	ACTTTCCAATGAGCATTCCGAGGA	This study
CNC-7/Fw	CAGGTTCAATGCAGTATGGCTATGG	This study
CNC-7/Rv	GGACGGTACATTCCCATACC	This study
FIPR-22/23 Fw	GCTGAAGCTCCACACATCC	[19]
FIPR-22/23 Rv	TATCCCATTCCTCCGTATCC	[19]

^a^ The letters Fw and Rv in the primers names describe the orientation of the primers 5′ to 3′: F for forward (sense) and R for reverse (antisense); ^b^ the efficiency of primers was evaluated based on the slope of the standard curve constructed by a 10 fold-dilution series using the cDNA.
